# Emergence of NDM-5-Positive *Salmonella enterica* Serovar Typhimurium Monophasic Variant ST34 in Hong Kong During 2020–2025

**DOI:** 10.3390/microorganisms14102289

**Published:** 2026-10-08

**Authors:** Atalie Ching-Yee Tse, Garnet Kwan-Yue Choi, Hayes Kam-Hei Luk, Eric Kam-Tong Yip, Chi-Ka Cheng, Sandy Ka-Yee Chau, Ho-Leung Ng, Herman Tse, Sally Cheuk-Ying Wong, David Christopher Lung

**Affiliations:** 1 Department of Pathology, Queen Elizabeth Hospital, Hong Kong SAR, China; atalie.tse@ha.org.hk (A.C.-Y.T.); hayes.luk@ha.org.hk (H.K.-H.L.); wcy288@ha.org.hk (S.C.-Y.W.); 2 Department of Pathology, Hong Kong Children’s Hospital, Hong Kong SAR, China; 3 Department of Clinical Pathology, Tuen Mun Hospital, Hong Kong SAR, China; yipkt@ha.org.hk (E.K.-T.Y.); hl.ng1@ha.org.hk (H.-L.N.); 4 Department of Pathology, United Christian Hospital, Hong Kong SAR, China; cck936@ha.org.hk (C.-K.C.); chauky@ha.org.hk (S.K.-Y.C.); 5 Department of Laboratory Medicine, Khoo Teck Puat Hospital, Singapore 768828, Singapore

**Keywords:** non-typhoidal *Salmonella* spp., carbapenem, *bla*_NDM-5_ gene, ST34, multidrug resistance

## Abstract

The emergence of carbapenem-resistant non-typhoidal *Salmonella* species (CR-NTS) has been observed in recent years, causing increasing challenges in the clinical management and control of *Salmonella* infections. However, CR-NTS had not been comprehensively studied in Hong Kong previously. We retrieved archived non-typhoidal *Salmonella* isolates demonstrating carbapenem resistance from five public hospitals in Hong Kong during January 2020–June 2025. The isolates were characterised by NG-TEST CARBA-5 and subjected to whole-genome sequencing (WGS) and phylogenetic analysis. Clinical information of CR-NTS cases was also reviewed. Fourteen CR-NTS isolates (0.3%, 14/4404) were identified during the study period. All 13 CR-NTS isolates (one isolate could not be recovered) were NDM (New Delhi metallo-β-lactamase)-positive, with the first case identified in March 2023. WGS revealed six Group B *Salmonella* monophasic variants of *Salmonella enterica* serovar Typhimurium ST34 harbouring the *bla*_NDM-5_ gene. The most common plasmid replicon co-present among *bla*_NDM-5_-positive isolates was IncHI2–IncHI2A (63.6%, 7/11). Among cases of CR-NTS in our study, self-limiting gastroenteritis was the most common presentation (84.6%, 11/13); none presented with invasive infections; travel history was present in 7.7% (1/13). Phylogenetic analysis showed the genotypic relatedness of the ST34 isolates identified in Hong Kong to those on the Chinese mainland, with the possibility of local CR-NTS acquisition via imported food sources. Our findings highlighted the importance of a One Health approach to the control of CR-NTS and the need for continuous antimicrobial resistance surveillance of *Salmonella* spp. in Hong Kong.

## 1. Introduction

Non-typhoidal *Salmonella* species (NTS) remain one of the most prevalent pathogens in foodborne infections [1]. They are one of the four key global causes of diarrhoeal disease [2]. Each year, NTS causes over 90 million cases of infection worldwide [3], with peak incidence in rainy and warmer months [4]. In Hong Kong, NTS was the third most common pathogen causing food poisoning outbreaks in 2025, comprising 19% of all cases identified [5].

NTS has natural reservoirs in the gastrointestinal tracts of animals commonly consumed by humans [2]. The incubation period for NTS infection is around 6–72 h (usually 12–36 h), and most patients present with acute febrile gastroenteritis, which is typically self-limiting and does not require antibiotic treatment [2]. However, severe or invasive infections such as osteomyelitis, bacteraemia, and endovascular infections can occur in at-risk hosts, such as elderlies, infants, immunocompromised hosts, where antibiotics therapy should be used [6,7,8]. The first-line antibiotics currently recommended for invasive NTS infection are third-generation cephalosporins or fluoroquinolones [9]; if resistance to third-generation cephalosporin is detected, use of carbapenems should be considered [9].

Carbapenems are a class of broad-spectrum β-lactam antibiotics that includes doripenem, ertapenem, imipenem, and meropenem [10]. Clinical use of carbapenems is usually reserved for infections caused by Gram-negative organisms, including NTS, that are extended-spectrum beta-lactamase-producing, or not susceptible to other first-line antibiotics such as cephalosporins, fluoroquinolones, etc. [11,12]. However, carbapenem resistance has increasingly been seen in recent years [13]. The most common mechanism of carbapenem resistance is due to the production of carbapenemases, which inactivate carbapenems by hydrolyzing the β-lactam rings on carbapenems [13]. Genes of carbapenemases can be chromosomal or plasmid-encoded [11]; carbapenemase genes with reported major clinical significance include KPC (*Klebsiella pneumoniae* carbapenemase), OXA (oxacillinase), IMP (imipenemase), VIM (Verona integron-encoded metallo-β-lactamase), and NDM (New Delhi metallo-β-lactamase) [13]. Some of these genes are located within different integron structures connecting with mobile plasmids or transposons, which facilitate easy horizontal transfer of such carbapenemase-encoding genes between different bacteria in close proximity within hosts or in the environment [13,14].

The emergence of carbapenem-resistant non-typhoidal *Salmonella* species (CR-NTS) is inevitable. One of the earliest cases of CR-NTS, isolated from a perirectal surveillance culture, was reported in the United States (US) in 2011 and carried *bla*_NDM-1_ [15]; since then, sporadic cases of CR-NTS have been reported worldwide, including in Europe, Asia, Africa, etc. [16,17], and all five clinically significant carbapenemases mentioned above have been found among these isolates [13]. Such antimicrobial resistance creates increasing challenges in the clinical management and control of *Salmonella* infections.

While CR-NTS has been reported worldwide, CR-NTS in clinical samples has not been comprehensively studied in Hong Kong. In this study, we aim to describe the prevalence, genomic epidemiology, and clinical characteristics of CR-NTS in Hong Kong.

## 2. Materials and Methods

### 2.1. Study Design and Setting

A retrospective review of NTS isolated from clinical specimens during January 2020–June 2025 at five public hospitals in Hong Kong (Queen Eliza-beth Hospital, United Christian Hospital, Tuen Mun Hospital, Pok Oi Hospital, Tin Shui Wai Hospital) was performed using data from the Laboratory Information System. For patients with more than one clinical sample isolating NTS, only the first isolate was counted. At the hospital microbiology laboratory, the NTS isolates were subjected to serogrouping according to the Kauffman–White classification [18] using the slide agglutination method with *Salmonella* O antiserum (Remel, Basingstoke, UK; SSI Diagnostica, Hillerod, Denmark; Mast, Amiens, France) as per the manufacturer’s instructions [19]. Antibiotic cascade testing was also performed as elaborated in Section 2.2 below. CR-NTS, defined as resistance to ertapenem (minimum inhibitory concentration (MIC) ≥ 2 µg/mL according to the Clinical and Laboratory Standards Institute (CLSI)) [20], were recovered from −70 °C in Microbank 2D (Pro-lab diagnostics; Richmond Hill, ON, Canada), and subjected to matrix-assisted laser desorption/ionisation time-of-flight mass spectrometry (MALDI-TOF MS, IVD library version: DB-11758 MSP; Bruker, Bremen, Germany) to confirm bacterial speciation to the genus level before proceeding to further phenotypic and genotypic analysis.

### 2.2. Antimicrobial Susceptibility Testing and Characterisation of Carbapenem Resistance

**Figure 1:**
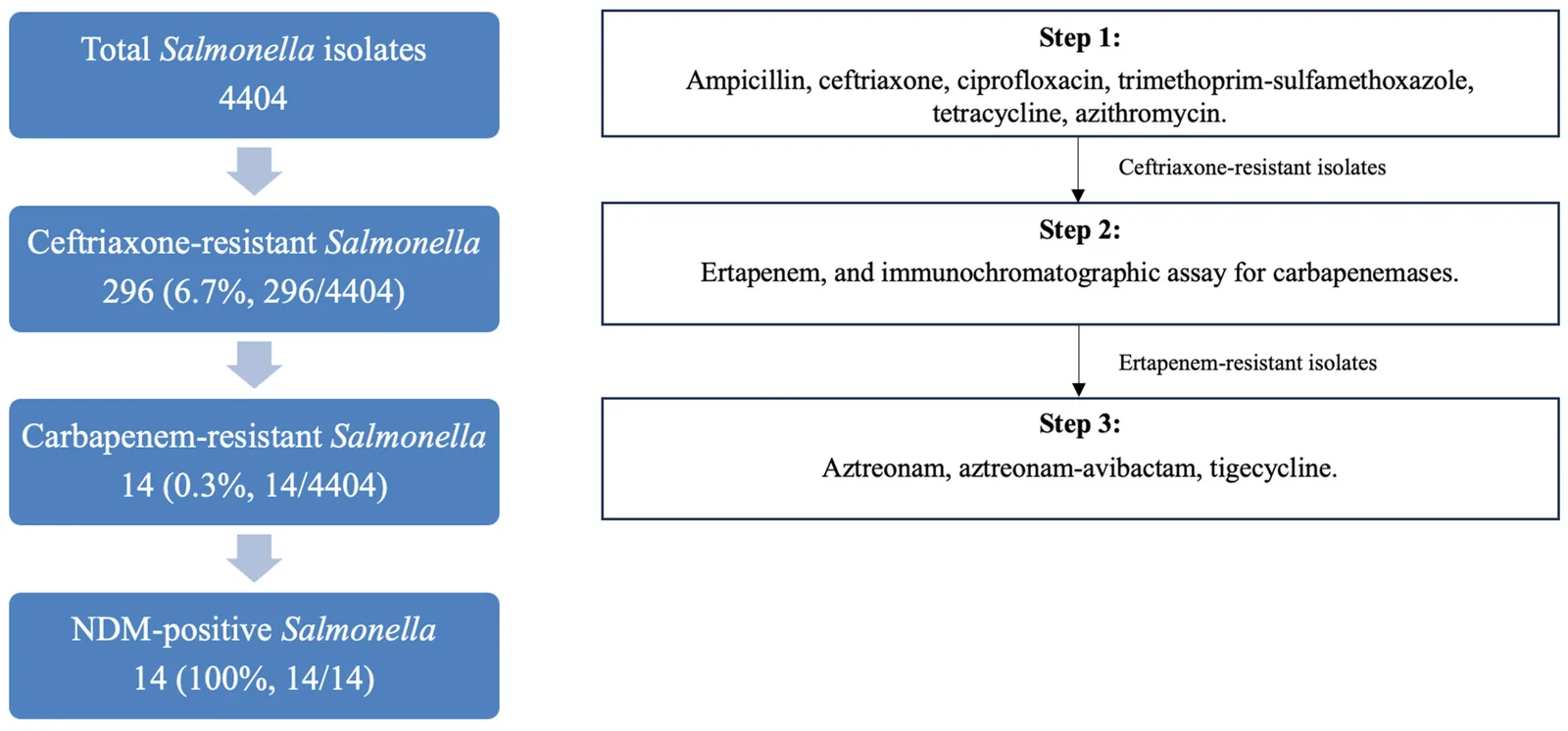
Antimicrobial susceptibility testing cascade and carbapenem resistance among clinical isolates of non-typhoidal *Salmonella* spp. collected from five public hospitals in Hong Kong between January 2020 and June 2025.

All NTS isolates were subjected to susceptibility testing cascade at the time of isolation, and isolates resistant to ceftriaxone were subjected to ertapenem disc diffusion testing according to the testing approach recommended by the CLSI [20] (Figure 1). For this study, NTS isolates resistant to ertapenem were recovered and steps 1–3 of antimicrobial susceptibility testing were performed (Figure 1). Susceptibility testing for ampicillin (10 µg, Oxoid, Basingstoke, UK), ciprofloxacin (5 µg, Oxoid, UK), trimethoprim-sulfamethoxazole (25 µg, Oxoid, UK), and tetracycline (30 µg, Oxoid, UK) was performed using the disc diffusion method; for ceftriaxone (30 µg, Oxoid, UK), azithromycin (15 µg, Oxoid, UK), and ertapenem (10 µg, Oxoid, UK) both the disc diffusion method and MIC determination using the gradient strip method were performed (Liofilchem, Roseto degli Abruzzi, Italy); and for aztreonam, aztreonam–avibactam, and tigecycline MIC determination using the gradient strip method was performed (Liofilchem, Italy). *Escherichia coli* ATCC 25922 was used as the quality control organism. Antimicrobial susceptibility testing methods and breakpoint interpretations were performed according to the CLSI if breakpoints were available [20]. For azithromycin, the breakpoint for *S. enterica* serovar Typhi was used (disc ≥ 13 mm, MIC ≤ 16 μg/mL) for interpretation [21].

All carbapenem-resistant *Salmonella* isolates were subjected to multiplex immunochromatographic assay NG-TEST CARBA-5 (NG Biotech, Guipry-Messac, France) for the detection of KPC, OXA-48-like, VIM, IMP, and NDM at the time of isolation, and this was repeated in our study according to the manufacturer’s instructions [22]. In brief, three colonies on blood agar were suspended in microtubes containing 150 µL of extraction buffer, and 100 µL of the homogenised mixture was added to the NG-TEST CARBA-5 test kit. Results were read and interpreted 15 min after the addition of the mixture [22].

### 2.3. Whole-Genome Sequencing and Phylogenetic Analysis of Carbapenem-Resistant Non-Typhoidal Salmonella

Genomic DNA from carbapenem-resistant isolates was extracted using a DNeasy Blood & Tissue Kit (Qiagen, Hilden, Germany), followed by library preparation using Illumina DNA Prep (Illumina, San Diego, CA, USA). Sequencing of the pooled library was performed using a MiSeq sequencer (Illumina) with paired-end 300 bp reads. FastQC (v0.12.1) was used to assess raw and trimmed read quality prior to assembly [23]. The low-quality bases in the paired-end reads were filtered and trimmed using Trimmomatic v0.40 [24]. Quality-trimmed reads were then de novo assembled using SPAdes v4.2.0 [25]. Assembly quality metrics were evaluated using QUAST (v5.2.0) [26]. Assemblies were retained for downstream bioinformatic analyses only if they met the following pre-defined quality acceptance thresholds, which are taken as reference from Qualibact (N50: ≥62,000 bp; Contigs: ≤200; GC Content: 51–53%; Length: 4.4–5.9 Mbp) [27]. The whole-genome sequencing (WGS) project has been deposited in NCBI BioProject database under accession number PRJNA1470784. Individual Sequence Read Archive (SRA) accession numbers (SRR38847552–SRR38847564) are provided in Table 1.

### 2.4. Multi-Locus Sequence Typing (MLST) and In Silico Serotype Prediction

The WGS data was used to determine the multi-locus sequence type of each isolate. Allele numbers and sequence types (STs) were assigned by querying the *Salmonella* spp. database in PubMLST (https://pubmlst.org/organisms/salmonella-spp (accessed on 24 November 2025)) [28,29]. In silico serotype prediction was performed using the SISTR (*Salmonella* In Silico Typing Resource) [30] analysis available in Pathogenwatch (https://pathogen.watch/ (accessed on 9 January 2026)) [31].

### 2.5. Phylogenetic Analysis

The core genome MLST (cgMLST) phylogenetic tree was constructed using the Genome Comparator tool [32] from the Bacterial Isolate Genome Sequence Database (BIGSdb) [32] available on the PubMLST website. Selected isolates of ST34 and ST2379, which differ from ST34 by a single allele, and scheme cgMLST v2 (Enterobase) were used to generate the Neighbour-Joining tree with the default parameters. The isolates selected for the cgMLST were from clinical, food and animal sources and were restricted to isolates with complete information on country, year and source in the PubMLST database. Single-nucleotide polymorphism (SNP) analysis was performed using snippy v4.60 (https://github.com/tseemann/snippy (accessed on 25 November 2025)). *Salmonella enterica* strain LT2 (accession number NC_003197.2) was selected as the reference genome. Selected clinical and animal ST34 *bla*_NDM-5_-positive CR-NTS isolates detected in China in recent years were used as comparator genomes [33,34,35,36,37,38,39,40,41,42]. A maximum-likelihood phylogeny was constructed from the SNP data using IQ-TREE v3.1.2 [43]. The optimal nucleotide substitution model was automatically determined using ModelFinder integrated within IQ-TREE 3 according to the Bayesian Information Criterion (BIC). For the core-SNP matrix, the K3Pu + ASC model was selected. The ascertainment bias correction (+ASC) was applied to correct for the absence of constant sites in the alignment. Branch and node support was evaluated using 1000 replicates of the SH-like approximate likelihood ratio test (SH-aLRT) (-alrt 1000). Nodes with SH-aLRT support values ≥ 80% were considered statistically well-supported.

### 2.6. Review of Phenotypic and Genotypic Antibiotics Resistance Patterns

Acquired antimicrobial resistance genes and chromosomal mutations were identified using ResFinder v4.7.2 (https://genepi.food.dtu.dk/resfinder, accessed on 13 March 2026) with default settings and *Salmonella* spp. as the reference species [44]. The results were then compared to the phenotypic results from antimicrobial susceptibility testing. In silico detection and typing of plasmids were performed using PlasmidFinder 2.1 with the Enterobacterales database (https://cge.food.dtu.dk/services/PlasmidFinder, accessed on 4 November 2025) [45,46].

### 2.7. Retrieval of Clinical Data

Demographic data on the patients, including age, gender, symptoms and signs of presentation, travel history within seven days before symptom onset, social history, investigation results, clinical progress, and outcome, were retrieved by reviewing electronic medical records in the Clinical Management System.

### 2.8. Statistical Analysis

Statistical analysis was performed using SPSS version 24 (SPSS, Chicago, IL, USA). Categorical variables were entered, and calculations of frequencies and percentages were performed. No inferential statistical tests were performed.

## 3. Results

**Figure 2:**
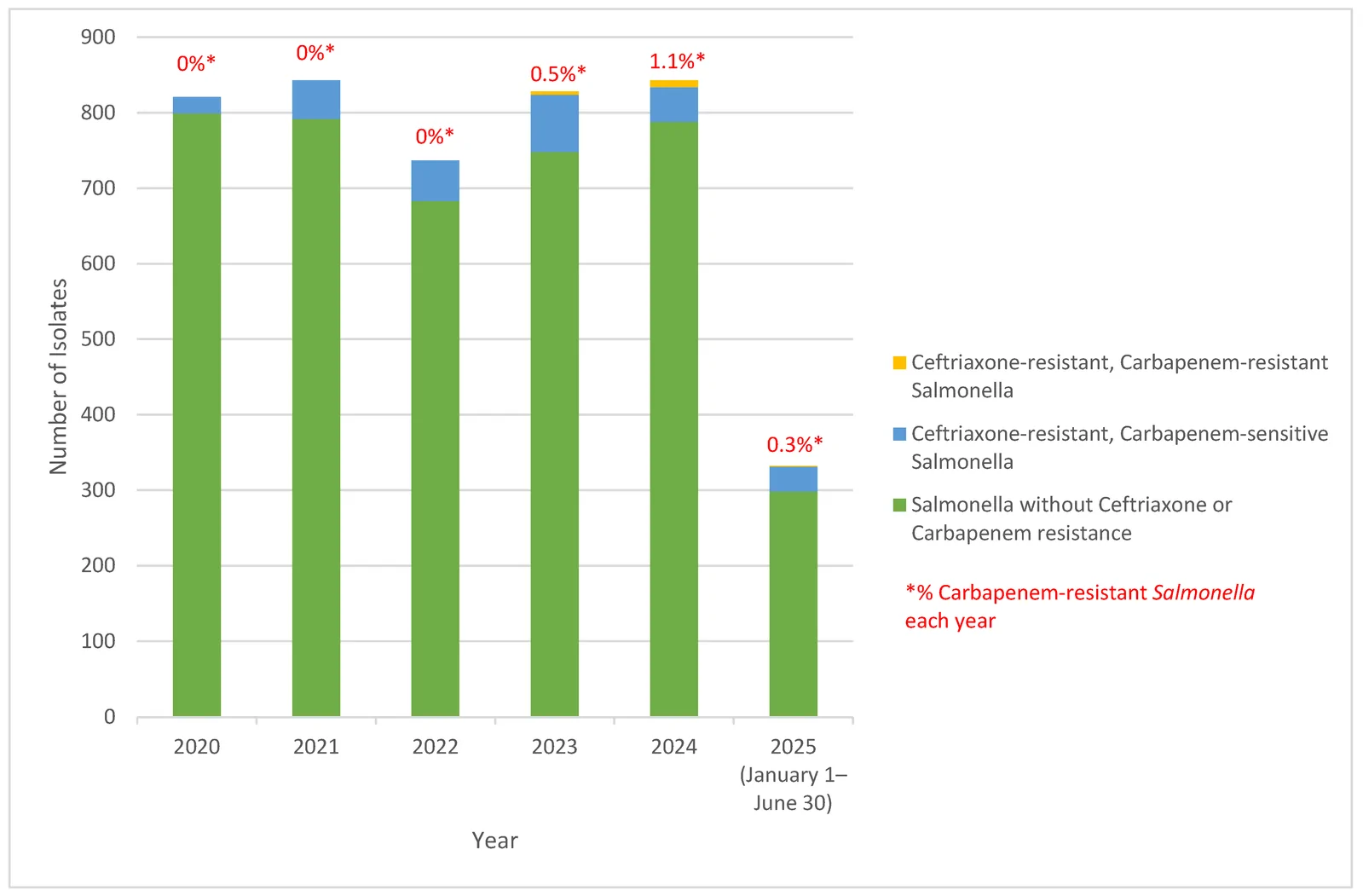
Non-typhoidal *Salmonella* spp. isolated in clinical specimens from five public hospitals in Hong Kong between January 2020 and June 2025. The overall prevalence of CR-NTS was 0.3% in the study period, with CR-NTS detected every year from 2023.

A total of 4404 non-duplicating NTS isolates were identified between January 2020 and June 2025 across five public hospitals in Hong Kong. Among all NTS isolates, ceftriaxone resistance was found in 6.7% of isolates (296/4404), of which 14 were also resistant to carbapenem (0.3%, 14/4404) (Figure 1 and Figure 2).

### 3.1. Characterisation of Carbapenem-Resistant Non-Typhoidal Salmonella

Among the 14 CR-NTS isolates, 13 were isolated from stool and one was isolated from rectal swab specimens; all tested positive for NDM by immunochromatographic assay. Of the 14 carbapenem-resistant isolates, one could not be recovered, and 13 isolates were subjected to further analysis. All the assemblies of the 13 isolates met the pre-defined criteria (N50: ≥62,000 bp; Contigs: ≤200; GC content: 51–53%; length: 4.4–5.9 Mbp). Of the 13 CR-NTS isolates, 11 (84.6%, 11/13) belonged to Group B, one each to Groups C and E (Table 1).

**Table 1:** Genotypic data of 13 NDM-positive non-typhoidal *Salmonella* isolates collected in Hong Kong between January 2020 and June 2025.

*Salmonella* Isolate No.	Group	Genotypic Antigenic Profile	MLST	Plasmid Type (s)	*bla*_NDM_ Variant	Accession Number
SAL01	*Salmonella* Group B	Stanley	29	IncHI2–IncHI2A	*bla*_NDM-5_	SRR38847564
SAL02	*Salmonella* Group B	1,4,[5],12:i:-	34	IncHI2–IncHI2A; IncI1-I(Alpha)	*bla*_NDM-5_	SRR38847563
SAL03	*Salmonella* Group B	1,4,[5],12:i:-	34	IncHI2–IncHI2A; IncFII	*bla*_NDM-5_	SRR38847559
SAL04	*Salmonella* Group B	1,4,[5],12:i:-	34	IncHI2–IncHI2A; IncQ1	*bla*_NDM-5_	SRR38847558
SAL05	*Salmonella* Group B	Indiana	17	IncHI2–IncHI2A; IncN	*bla*_NDM-9_	SRR38847557
SAL06	*Salmonella* Group B	1,4,[5],12:i:-	34	IncI1-I(Alpha); IncQ1; IncX1	*bla*_NDM-5_	SRR38847556
SAL07	*Salmonella* Group B	Typhimurium	19	IncI1-I(Alpha); IncQ1; Col440I; p0111	*bla*_NDM-5_	SRR38847555
SAL08	*Salmonella* Group E	E1:l,v:-	654	ColpVC	*bla*_NDM-5_	SRR38847554
SAL09	*Salmonella* Group C	Rissen	469	IncFII(pCoo); IncY; Col(pHAD28);	*bla*_NDM-1_	SRR38847553
SAL10	*Salmonella* Group B	1,4,[5],12:i:-	2379	IncHI2–IncHI2A; IncQ1	*bla*_NDM-5_	SRR38847552
SAL11	*Salmonella* Group B	Stanley	29	IncHI2–IncHI2A	*bla*_NDM-5_	SRR38847562
SAL12	*Salmonella* Group B	1,4,[5],12:i:-	34	IncHI2–IncHI2A; IncQ1; Col440I	*bla*_NDM-5_	SRR38847561
SAL13	*Salmonella* Group B	1,4,[5],12:i:-	34	IncI1-I(Alpha); IncQ1; Col(pHAD28); p0111	*bla*_NDM-5_	SRR38847560

All 13 isolates showed resistance to multiple antimicrobials (Table 2). All 13 were resistant to ampicillin, ceftriaxone, ertapenem, and tetracycline. The ertapenem MIC ranged from 2 to 32 µg/mL. ResFinder analysis of antibiotic-resistant genes confirmed that 11 strains (84.6%, 11/13) carried the *bla*_NDM-5_ gene, while the remaining two strains carried the *bla*_NDM-1_ and *bla*_NDM-9_ genes, respectively, which is compatible with the immunochromatographic result (Table 1). Susceptibility to ciprofloxacin and trimethoprim-sulfamethoxazole were 7.7% (1/13) and 23.1% (3/13). For ciprofloxacin, all isolates with intermediate susceptibility harboured the *qnrS1* gene, while the resistant isolate carried *aac*(*6′*)*-Ib-cr* (SAL05). Good concordance of susceptibility results was noted between azithromycin disc diffusion test and MIC. The only azithromycin-resistant isolate (SAL05) harboured the *mph*(A) gene showed resistance with an MIC of 256 µg/mL. Susceptibility testings towards antibiotics commonly used for carbapenem-resistant Enterobacterales (CREs) including aztreonam, aztreonam–avibactam, and tigecycline were also performed although no CLSI breakpoints are available. Three isolates (23.1%, 3/13) with a high MIC (≥32 µg/mL) towards aztreonam all harboured the *bla*_CTX-M-55_ gene, while the other CR-NTS showed an aztreonam MIC of ≤0.125 µg/mL. All 13 isolates demonstrated a relatively low MIC range towards aztreonam–avibactam (<0.016–0.19 µg/mL) and tigecycline (0.25–1 µg/mL). None of the 13 isolates carried the *mcr* genes. There was high concordance between resistant gene profiles and phenotypical resistance, and the other antibiotic-resistant genes identified are listed in Supplementary Table S1. Among the isolates with the *bla*_NDM-5_ gene, the most common co-present plasmid replicon identified was IncHI2–IncHI2A (63.6%, 7/11); only one isolate carried the IncFII plasmid (Table 1).

**Figure 3:**
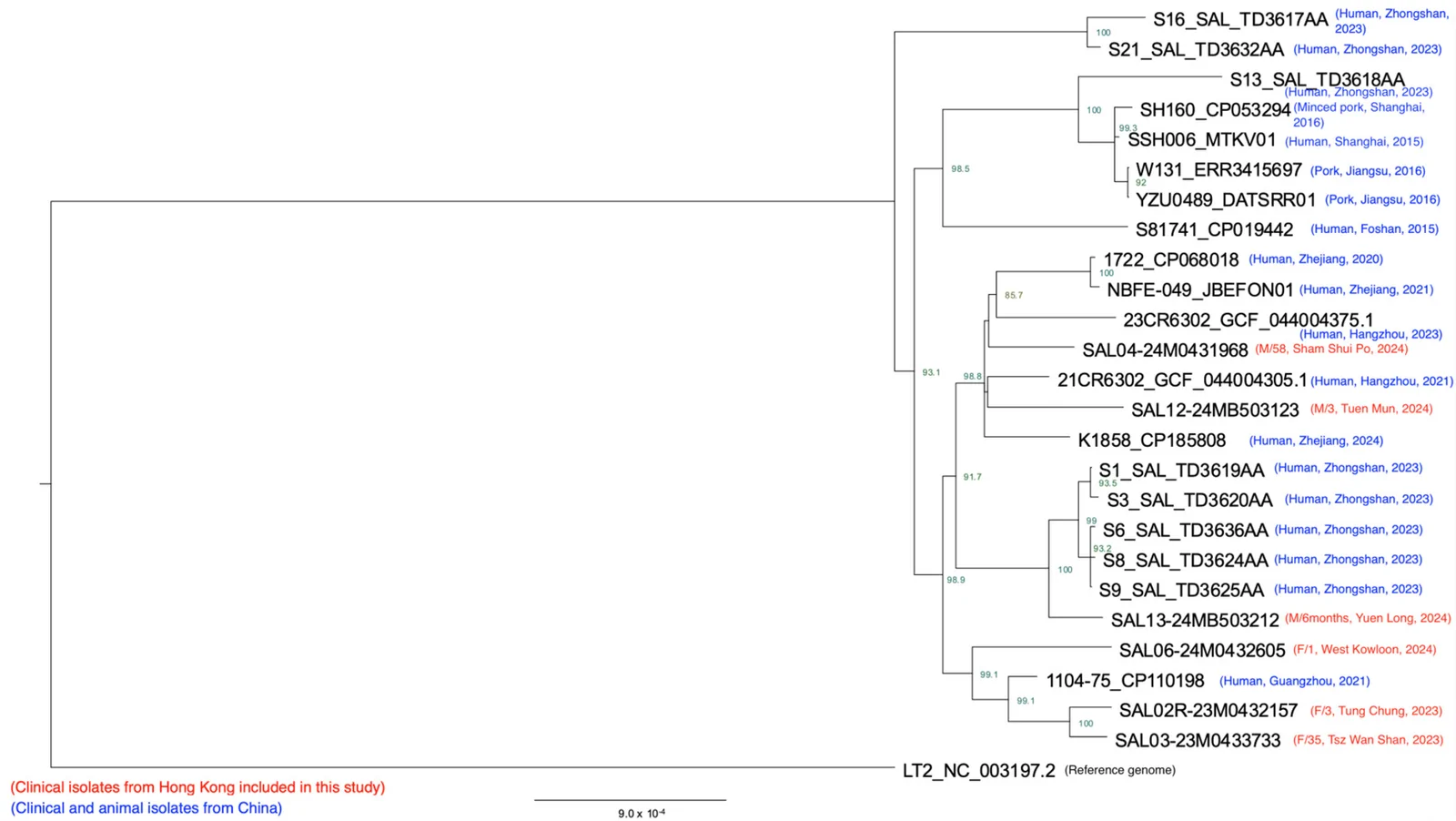
Phylogenetic tree of *bla*_NDM-5_-positive Salmonella enterica serovar Typhimurium monophasic variant isolates ST34 from this study, and selected clinical and animal isolates from China detected in recent years [33,34,35,36,37,38,39,40,41,42]. The tree was constructed using IQ-TREE 3 under the K3Pu + ASC substitution model with 1000 SH-aLRT replicates for branch support (node values ≥ 80% are shown). 23–126 SNP differences were demonstrated among these isolates.

Genotypic analysis by WGS revealed six Group B *Salmonella* isolates (46.2%, 6/13), belonging to the monophasic variant 1,4,[5],12:i:- of *Salmonella enterica* serovar Typhimurium ST34, harbouring the *bla*_NDM-5_ gene responsible for carbapenem resistance (Table 1). Phylogenetic analysis comparing the six ST34 isolates from our study with other human and animal ST34 isolates found in China and carrying the *bla*_NDM-5_ gene demonstrated a difference of 23–126 SNPs (Figure 3). When compared with global ST34 and ST2379 strains, it was noted that our ST34 and ST2379 isolates closely clustered with isolates from China (Supplementary Figure S1).

### 3.2. Clinical Epidemiology and Clinical Data of NDM-Positive Salmonella Cases

The first case of CR-NTS in our study emerged in March 2023, with three additional cases identified later in 2023 (0.5%, 4/828). Nine cases were detected in 2024 (1.1%, 9/843), and one case was detected up to 30 June 2025 (0.3%, 1/332) (Figure 2). Excluding the case of CR-NTS, which could not be recovered, 12 out of 13 cases (92.3%, 12/13) were detected between March and October, with a peak incidence of three cases in June (23.1%, 3/13).

Patients with NDM-positive NTS ranged in age from five months to 85 years (median, 36 years); five were children aged three years or younger (38.5%, 5/13) (Table 3). Self-limiting gastroenteritis was the most common presentation, occurring in 84.6% of cases (11/13), and fever was present in 69.2% (9/13) (Table 3). None developed invasive infection. One elderly patient (SAL08) had asymptomatic carriage of CR-NTS, detected from a rectal swab for CRE screening upon hospital admission. One patient (7.7%, 1/13) reported recent travel to the Chinese mainland within one week before symptom onset (Table 3). Eight patients (61.5%, 8/13) received antibiotic therapy (Table 3). Three patients (SAL05, SAL06, SAL13) received ceftriaxone with no alteration of antibiotics after resistance to ceftriaxone was reported (Table 3). None of these patients developed complications from the infection and all patients have recovered fully.

## 4. Discussion

This is the first report to describe the emergence of CR-NTS among clinical isolates in Hong Kong with a specific focus on their phenotypic and genomic characteristics. The current study demonstrated that CR-NTS first emerged in Hong Kong in 2023, with over 90% detected from March to October, corresponding to late spring and summer months in Hong Kong. More specifically, over half of the cases (7/13, 53.8%) presented between April and June, when the average temperature and relative humidity are over 25 °C and 80% in Hong Kong [47]. According to the Hong Kong Centre for Health Protection 2016–2025 data, food poisoning outbreaks peaked in May and June, with *Salmonella* spp. being one of the most common bacteria isolated [48]. It is observed that the cases of CR-NTS infections included in our study also showed similar seasonal patterns.

The emergence of CR-NTS in 2023 was also observed in nearby areas of Hong Kong. Routine surveillance performed by the Centers for Disease Control and Prevention in Southern China first detected clusters of community-acquired CR-NTS infections in mid-2023 [33]. Further genomic studies revealed that the majority of CR-NTS in China carried the *bla*_NDM_ gene, particularly *bla*_NDM-5_ since 2023 [33]. In contrast, worldwide analysis of CR-NTS isolates reported that *bla*_OXA-48_ gene was predominant in countries such as the United Kingdom (UK), France, South Africa [33]. The CR-NTS isolated in our cohort all carried *bla*_NDM_, with *bla*_NDM-5_ being most common. *bla*_NDM-5_, differing from *bla*_NDM-1_ by two amino acid substitutions at positions 88 and 154 that result in higher resistance levels, was first reported in 2011 in the UK in a patient with a multidrug-resistant *E. coli* ST648 strain, carried by an IncF plasmid, who had had a recent history of hospitalisation in India [49]. A rapid increase in *bla*_NDM-5_ in clinical isolates of Enterobacterales has been noted globally since 2015, with it replacing *bla*_NDM-1_ as the predominant strain in some areas [50], including China [51] and Europe [52]. A report written by the European Centre for Disease Prevention and Control speculated that most acquisitions were travel-related, with more than 80% of NDM-5-positive *E. coli* being detected in persons with travel and/or hospitalisation outside of the European Union and European Economic Area, mainly in Africa and Asia, within the six months before the sampling [52]. Concerningly, NDM-5-positive Enterobacterales have been reported in retail meats [53] and companion animals [54], suggesting potential bidirectional transmission across these different reservoirs, which raised significant concerns from a One Health perspective. While *E. coli* is the predominant carrier of *bla*_NDM-5_, findings of *bla*_NDM-5_ have been reported in other Enterobacterales, including NTS [36,55]. This could be due to the association of *bla*_NDM_ with plasmids, especially IncX3, IncFII, and to a lesser extent IncHI2, that are transmissible between Enterobacterales [34,39,53,56,57]. Among our NDM-5-positive NTS, the predominant co-present plasmid type was IncHI2–IncHI2A (63.6%, 7/11), and only one isolate (SAL03) carried the IncFII plasmid (9.1%, 1/11). Other than *bla*_NDM_, many other resistance genes were identified in our CR-NTS isolates, including beta-lactamase genes such as *bla*_TEM_, *bla*_CTX_, *bla*_OXA-10_, and *qnrS1*, leading to ciprofloxacin non-susceptibility. All isolates were resistant to tetracycline, and 76.9% (10/13) were resistant to trimethoprim-sulfamethoxazole, further limiting the oral options. Fortunately, many have remained susceptible to azithromycin, and none of the isolates carried *mcr* genes, unlike the findings of an earlier study [16].

The dominating sequence type of our study’s isolates, ST34, is a relatively new ST found in *S.* Typhimurium and its monophasic variant. However, ST34 has raised increasing global concerns regarding its spread and multidrug resistance (MDR) in recent years. Earlier reports showed that ST34 emerged in the 1990s in Europe when the dominating clone was still ST19. Since then, ST34 has spread to various European countries, the US, Australia, and China, replacing the traditional ST19 clone as the dominant clone [16,58]. In fact, ST34 is frequently isolated from food animals. A study done in French pig farms and slaughterhouses in 2014–2019 found that a majority of *S.* Typhimurium and its monophasic variant belonged to ST34 [59]. In Shenzhen, a Chinese city in close proximity to Hong Kong, a significant rise in *S.* Typhimurium and its monophasic variant was also observed in 2007–2023 across clinical, food, and occupational samples, of which over 97% belonged to ST34 [60]. Its successful spread and worldwide prevalence may be attributed to its MDR nature. A study involving analysis of 196 *S.* Typhimurium isolates found in China from 2007 to 2019 detected 100% resistance to ampicillin, amoxicillin/clavulanic acid, and tetracycline in ST34 isolates, mostly isolated from pig and human specimens, which is consistent with the higher use of antibiotics in these populations [61]. Comparing to other sequence types, two specific virulence features were reported to contribute to the emergence of ST34. Firstly, tolerance genes to heavy metals in ST34 led to its better survival and spread in animals, especially food animals like pigs, where copper and zinc supplements are commonly used to enhance animal growth [16,62,63]. Secondly, a stronger biofilm-forming ability was detected in ST34 isolates from patients in Southern China [64]. Stronger biofilm formation has been demonstrated to improve bacterial survival and the evolution of MDR, leading to an increased risk of human infections and complications [16].

Our data revealed that 84.6% of our isolates (11/13) carried the *bla*_NDM-5_ gene and over half of the isolates (53.8%, 7/13) belonged to ST34 or its closely related sequence type ST2379 (Table 1). Phylogenetic analysis shows that these strains are closely clustered with isolates reported from China (Figure 3 and Figure S1), but genetic clustering cannot confirm epidemiological transmission. Only one patient (SAL01) in our cohort (7.7%, 1/13) reported recent travel within one week prior to symptom onset (Table 3), indicating that most patients in our study likely acquired their infection locally. According to the Environment and Ecology Bureau in Hong Kong, the majority of fresh food, especially pork, beef, and chicken, was imported from the Chinese mainland [65,66]. A literature search in PubMed, including NDM-positive NTS in food animals from China reported in English peer reviewed journals, revealed that multiple studies done in the past ten years have also isolated NDM-positive CR-NTS from retail meat sourced in China, with *bla*_NDM-5_ being more commonly found in the recent studies (Table 4) [38,41,42,67,68,69,70,71,72]. Although another local study done in Hong Kong in 2022–2023 did not identify any CR-NTS, it did detect multidrug-resistant NTS from retail meats that showed phylogenetic relevance to clinical isolates, suggesting highly possible and concerning transmission from retail meats to humans [73]. Given the increasing detection of NDM-positive *Salmonella* from clinical isolates and food animal products in China, there is a possibility that these CR-NTS could have been acquired via imported food sources due to Hong Kong’s high reliance on imported food from the Chinese mainland, with subsequent cross-contamination between raw food and kitchen utensils during food preparation or faecal–oral route transmission.

From a clinical perspective, the emergence of CR-NTS in Hong Kong is concerning and warrants a review of antibiotic choices in managing *Salmonella* infections. Apart from carbapenem, these CR-NTS isolates in our studies also demonstrated resistance to multiple classes of antibiotics (Table 2). The latest edition of the Hong Kong antimicrobial chemotherapy guidelines recommends oral azithromycin as the antibiotic of choice for moderate-to-severe bacterial gastroenteritis in immunosuppressed patients and patients with traveller’s diarrhoea [74]. More than 90% of the CR-NTS isolates in our study remained susceptible to azithromycin (Table 2) when a MIC breakpoint of 16 μg/mL for *S.* Typhi was adopted to interpret azithromycin resistance, which is supported by a study proposing the same MIC as an epidemiological cutoff value for wild-type *S. enterica* [21]. The lower rates of resistance towards azithromycin in *Salmonella* spp. compared to other antibiotics may be attributed to the restricted use of azithromycin in food animals [75]. Nevertheless, increasing azithromycin resistance has been noted in NTS in recent years, commonly mediated by resistance genes *mph*(A), *erm*(42), *erm*(B), and *ramAp* located in mobile genetic elements which can be easily disseminated [76].

Although there was no invasive *Salmonella* infection in the current study cohort, invasive infection due to CR-NTS may occur when the incidence gradually increases, where azithromycin may not be a suitable drug. Alternative management options include antibiotics commonly used to treat CRE infections such as aztreonam, aztreonam–avibactam, and tigecycline; use of these antibiotics should be dependent on an individual isolate’s susceptibility (Table 2).

While no significant revision of clinical management guidelines for gastroenteritis is needed at the moment, we should be aware of the emergence of CR-NTS in Hong Kong, coupled with antibiotic susceptibility surveillance among NTS to curb the spread of resistant strains.

### 4.1. Future Directions

Previously the literature has suggested the horizontal transfer of carbapenemase-encoding plasmids between Enterobacterales as a mechanism for *Salmonella* spp. to acquire carbapenem resistance [33,38]. According to data from the Centre for Food Safety in Hong Kong, routine surveillance on food antimicrobial resistance between 2022 and 2025 has persistently detected CRE in over 20% of raw meat [77]. Therefore, further analysis and comparison of bacterial plasmids found in animal and human isolates may provide insights into the acquisition of carbapenemase genes in NTS in Hong Kong.

The limited number of antibiotic options to treat CR-NTS infection is worrying, especially if invasive infection arises. Clinical data and antibiotics susceptibility patterns of future CR-NTS isolates need to be collected for further analysis to establish evidence for recommended treatment in invasive CR-NTS infection.

### 4.2. Call to Action

The emergence of CR-NTS in Hong Kong signifies a silent spread of carbapenem resistance among NTS. Since the source of infection is likely food-related, public education of food hygiene is the most effective method to prevent human infections. Hong Kong has a comprehensive antimicrobial resistance (AMR) surveillance system, established in 2016 and based on the Global Antimicrobial Resistance Surveillance System developed by the World Health Organization (WHO) [78]. Under the One Health framework, the Hong Kong AMR surveillance system includes both human and animal AMR statistics [79]. Currently, the AMR surveillance for both local and imported fresh produce sold in Hong Kong includes three specific bacteria, *Staphylococcus aureus*, *Enterococcus*, and *E. coli*, where carbapenem-resistant *E. coli* has been demonstrated in cattle, pig, and chicken [79]. *Salmonella* spp. should also be considered for inclusion in the AMR surveillance system, as MDR *Salmonella* spp. has been listed as one of the pathogens in the WHO updated Bacterial Priority Pathogens List in 2024, and is classified as critical and a high priority for being carbapenem-resistant and fluoroquinolone-resistant, respectively [80].

The AMR patterns among *Salmonella* isolates in hospital and community settings should be reviewed regularly, which can guide the modification and updating of clinical guidelines on the empirical treatment of gastroenteritis and specific treatment for *Salmonella* gastroenteritis. Since the spread of carbapenem-resistant *Salmonella* is likely a regional phenomenon, collaboration with neighbouring territories in terms of control, surveillance, and treatment would be necessary.

### 4.3. Limitations

The clinical laboratories involved in our study currently adopt the cascade testing approach according to the CLSI guidelines for the detection of CR-NTS. Under this screening method, there is a possibility of missing ceftriaxone-susceptible but carbapenem-resistant strains, e.g., strains carrying carbapenemase with weak activity against broad-spectrum cephalosporins such as OXA-48-like enzymes [81,82]. Future studies with universal screening for carbapenem resistance regardless of ceftriaxone susceptibility should be done to investigate the potential gap in the cascade approach.

Moreover, despite analysing the plasmids carrying the *bla*_NDM_ gene in our cohort of isolates, conjugation assays were not performed in this study and short read WGS alone constrained our ability in the full reconstruction of plasmid structures. Future studies integrating long-read sequencing technologies with functional conjugation assays can provide more insights on the source of resistance gene acquisition, and plasmid transmission between our isolates and other Enterobacterales.

Thirdly, our study only involved five regional public hospitals in Hong Kong. Further analysis of isolates from other public hospitals, private hospitals, and the veterinary sector could provide a more comprehensive view of the transmission dynamics and genomic epidemiology of CR-NTS in Hong Kong. Furthermore, *Salmonella* infections commonly present as self-limiting gastroenteritis where healthy individuals usually recover spontaneously. A considerable percentage of patients infected by CR-NTS might not have sought medical attention and therefore no stool specimens were saved for cultures. As a result, the actual incidence of CR-NTS infection may be underestimated.

Lastly, due to the retrospective nature of this study, only clinical notes were retrieved, which limits our understanding of patients’ epidemiological exposure and ability to draw well-supported conclusions on their route of CR-NTS acquisition. Moreover, the current study is a hospital-based study. We have limited access to food, animal, and environmental isolates; hence, we were unable to establish direct genomic relatedness to the clinical isolates in the current study.

## 5. Conclusions

This study describes the emergence of carbapenem-resistant non-typhoidal *Salmonella* in Hong Kong since 2023 and the isolates’ phenotypic and genotypic characteristics. All CR-NTS isolates in the study carried *bla*_NDM_, and phylogenetic analysis showed the genotypic relatedness of the ST34 isolates identified in Hong Kong to those in Chinese mainland. Local CR-NTS acquisition via imported food sources is a possibility; however, the specific route and source of introduction into Hong Kong requires further study. Phylogenetic analysis revealed widespread dissemination of different strains of CR-NTS which is unlikely related to a single point source. There is an urgent need to include *Salmonella* spp. in the AMR surveillance system in Hong Kong.

## Figures and Tables

**Table 2:** Antibiotic susceptibility pattern of NDM-positive non-typhoidal *Salmonella* spp. detected in Hong Kong.

No.	AMP	CIP	SXT	TET	AZI		CRO		ETP		ATM	ATM-AVI	TGC
	S/I/R	S/I/R	S/I/R	S/I/R	S/I/R	MIC	S/I/R	MIC	S/I/R	MIC	MIC	MIC	MIC
SAL01	R	I	R	R	S	3	R	32	R	12	0.125	0.125	0.38
SAL02	R	I	S	R	S	3	R	32	R	4	96	0.064	0.38
SAL03	R	I	S	R	S	12	R	32	R	12	32	0.047	0.25
SAL04	R	I	R	R	S	4	R	32	R	2	0.125	0.094	0.5
SAL05	R	R	R	R	R	256	R	32	R	8	64	0.19	1
SAL06	R	S	S	R	S	3	R	32	R	32	0.047	0.064	0.5
SAL07	R	I	R	R	S	3	R	32	R	16	<0.016	<0.016	0.25
SAL08	R	I	R	R	S	4	R	32	R	2	0.047	0.047	0.75
SAL09	R	I	R	R	S	2	R	32	R	2	0.047	0.047	0.5
SAL10	R	I	R	R	S	3	R	32	R	3	0.047	0.047	0.25
SAL11	R	I	R	R	S	2	R	32	R	2	0.047	0.064	0.38
SAL12	R	I	R	R	S	2	R	32	R	2	0.047	0.047	0.38
SAL13	R	I	R	R	S	3	R	32	R	8	0.047	0.064	0.75

AMP: Ampicillin; CIP: Ciprofloxacin; SXT: Trimethoprim-sulfamethoxazole; TET: Tetracycline; AZI: Azithromycin; CRO: Ceftriaxone; ETP: Ertapenem; ATM: Aztreonam; ATM-AVI: Aztreonam–avibactam; TGC: Tigecycline; R: Resistant; S: Susceptible; I: Intermediate; MIC: Minimum inhibitory concentration (μg/mL).

**Table 3:** Clinical data on 13 NDM-positive non-typhoidal *Salmonella* isolates detected in Hong Kong during January 2020–June 2025.

*Salmonella* Isolate No.	Year/Month	Age (Year)	Gender	Residential District	Travel History *	Fever	Diarrhoea	Vomiting	Abx	Abx Given
SAL01	2023/3	58	F	Yau Ma Tei	+	−	+	+	−	
SAL02	2023/6	3	F	Tung Chung	−	+	+	−	−	
SAL03	2023/9	35	F	Tsz Wan Shan	−	+	+	+	+	Augmentin IV
SAL04	2024/5	58	M	Sham Shui Po	−	+	+	+	+	Ceftriaxone IV, Levofloxacin PO
SAL05	2024/6	36	F	To Kwa Wan	−	+	+	+	+	Ceftriaxone IV
SAL06	2024/7	15 months	F	West Kowloon	−	+	+	−	+	Ceftriaxone IV
SAL07	2024/10	68	F	Tsz Wan Shan	−	+	+	+	+	Augmentin IV, PO
SAL08	2025/5	77	M	Diamond Hill	−	−	−	−	−	
SAL09	2023/8	85	F	Yau Tong	−	+	+	−	+	Azithromycin PO
SAL10	2024/6	52	M	Yuen Long	−	−	+	+	+	Augmentin IV, Cefotaxime IV, Azithromycin IV
SAL11	2024/1	5 months	M	Tin Shui Wai	−	−	+	−	−	
SAL12	2024/4	3	M	Tuen Mun	−	+	+	+	−	
SAL13	2024/4	6 months	M	Yuen Long	−	+	+	−	+	Ceftriaxone IV, Cefpodoxime PO

* Travel history within one week before symptom onset; Abx: Antibiotics.; +: Positive; −: Negative.

**Table 4:** Report of NDM-positive non-typhoidal *Salmonella* in retail meat from China.

Isolate Name	Year	Province	Serotype (Number of Strains)	Carbapenem Resistance Tested	Gene	Source	NDM Gene Location	Project Number	Accession Number	Reference
C629	2014	Shandong	*S.* Indiana (1)	Imipenem Meropenem	*bla*_NDM-1_	Chicken carcass	Plasmid	PRJNA320977	CP015724	[67]
SH160	2016	Shanghai	*S.* Typhimurium ST34 (1)	Imipenem	*bla*_NDM-5_	Minced pork	Plasmid	PRJNA625025	CP053294.1	[41]
W131	2016	Jiangsu	*S.* 1,4,[5],12:i:- ST34 (1)	Meropenem	*bla*_NDM-5_	Pork	Plasmid	PRJEB33347	ERR3415697	[42]
YZU0489	2016	Jiangsu	*S.* 1,4,[5],12:i:- ST34 (1)	Not specified	*bla*_NDM-5_	Pork	Not specified	PRJEB50816	DATSRR01	[38]
s202009-74	2020	Guangzhou	*S.* Indiana ST17 (1)	Imipenem Meropenem	*bla*_NDM-9_	Chicken	Transposon	PRJNA1101247	JBCDUB000000000	[68]
S202011-11	2020	Guangdong	*S.* Kentucky ST 198 (1)	Imipenem	*bla*_NDM-5_	Chicken	Chromosome	PRJNA1175019	CP173281	[69]
s202101-61	2021	Guangzhou	*S.* Indiana ST17 (1)	Imipenem Meropenem	*bla*_NDM-1_	Chicken	Transposon	PRJNA1101247	JBCDSE000000000	[68]
YZ21MCS4	2021	Yangzhou	*S.* Indiana ST17 (1)	Meropenem	*bla*_NDM-9_	Chicken	Chromosome	PRJNA787409	CP089313	[70]
22S130	2022	Hebei	*S.* Indiana ST17 (1)	Imipenem	*bla*_NDM-9_	Chicken	Plasmid	PRJNA1379270	JBYOGF000000000	[71]
S026	2024	Shandong	*S*. Kentucky ST198 (1)	Imipenem	*bla*_NDM-5_	Chicken	Chromosome	PRJNA1267742	Not specified	[72]
S029, S094	2024	Shandong	*S*. Indiana ST17 (2)	Imipenem	*bla*_NDM-5_	Chicken	Chromosome	PRJNA1267742	Not specified	[72]
S064, S130	2024	Shandong	*S*. Indiana ST17 (2)	Imipenem	*bla*_NDM-5_	Chicken	Plasmid	PRJNA1267742	Not specified	[72]
S135	2024	Shandong	*S.* Iidikan ST1561 (1)	Imipenem	*bla*_NDM-5_	Chicken	Plasmid	PRJNA1267742	Not specified	[72]

## Data Availability

The original contributions presented in this study are included in this article. Further inquiries can be directed to the corresponding author.

## Supplementary Materials

The following supporting information can be downloaded at: https://www.mdpi.com/article/10.3390/microorganisms14102289/s1: Figure S1: Phylogenetic analysis of *Salmonella enterica* serovar Typhimurium and its monophasic variant isolates ST34 and ST2379; Table S1: Antibiotic resistance genes detected in the 13 clinical carbapenem-resistant non-typhoidal *Salmonella* isolates included in this study.

## Abbreviations

The following abbreviations are used in this manuscript:

NTS	Non-typhoidal *Salmonella* species
CR-NTS	Carbapenem-resistant non-typhoidal *Salmonella* species
WGS	Whole-genome sequencing
KPC	*Klebsiella pneumoniae* carbapenemase
OXA	Oxacillinase
IMP	Imipenemase
VIM	Verona integron-encoded metallo-β-lactamase
NDM	New Delhi metallo-β-lactamase
MIC	Minimum inhibitory concentration
CLSI	Clinical and Laboratory Standards Institute
MLST	Multi-Locus Sequence Typing
ST	Sequence type
BIC	Bayesian Information Criterion
+ASC	Ascertainment bias correction
SH-aLRT	SH-like approximate likelihood ratio test
cgMLST	Core genome MLST
SNP	Single-nucleotide polymorphism
CRE	Carbapenem-resistant Enterobacterales
MDR	Multidrug resistance
AMR	Antimicrobial resistance
WHO	World Health Organization

## References

[1] Kumar G., Kumar S., Jangid H., Dutta J., Shidiki A. (2025). The rise of non-typhoidal Salmonella: An emerging global public health concern. Front. Microbiol..

[2] World Health Organization (2018). Salmonella (Non-Typhoidal). https://www.who.int/news-room/fact-sheets/detail/salmonella-(non-typhoidal).

[3] Ranjan A., Chandna M., Stevens N.J., Kandil J., Dinh B., Kuhn M., Mian N., Tran B., Hamid A., Kim P. (2026). Salmonella Infections: Global Trends and Emerging Challenges. Microorganisms.

[4] Flores Monter Y.M., Chaves A., Arellano-Reynoso B., Lopez-Perez A.M., Suzan-Azpiri H., Suzan G. (2021). Edaphoclimatic seasonal trends and variations of the Salmonella spp. infection in Northwestern Mexico. Infect. Dis. Model..

[5] Or J., Choi Y.K. *Review of Food Poisoning Outbreaks Related to Food Premises and Food Business in 2025*; Food Safety Focus: Hong Kong SAR, China, 2026. https://www.cfs.gov.hk/english/multimedia/multimedia_pub/multimedia_pub_fsf_236_01.html.

[6] Centers for Disease Control and Prevention (2024). About Salmonella Infection. https://www.cdc.gov/salmonella/about/index.html.

[7] Bjorklund L., Mattisson Y., Blackberg A., Sunnerhagen T., Ljungquist O. (2024). A Population-Based Study on the Incidence, Risk Factors, and Outcome of Salmonella Bloodstream Infections in South Sweden 2012–2022. Infect. Dis. Ther..

[8] Marchello C.S., Birkhold M., Crump J.A., on behalf of the Vacc-iNTS Consortium Collaborators (2022). Complications and mortality of non-typhoidal salmonella invasive disease: A global systematic review and meta-analysis. Lancet Infect. Dis..

[9] Shane A.L., Mody R.K., Crump J.A., Tarr P.I., Steiner T.S., Kotloff K., Langley J.M., Wanke C., Warren C.A., Cheng A.C. (2017). 2017 Infectious Diseases Society of America Clinical Practice Guidelines for the Diagnosis and Management of Infectious Diarrhea. Clin. Infect. Dis..

[10] Hawkey P.M., Livermore D.M. (2012). Carbapenem antibiotics for serious infections. BMJ.

[11] Aurilio C., Sansone P., Barbarisi M., Pota V., Giaccari L.G., Coppolino F., Barbarisi A., Passavanti M.B., Pace M.C. (2022). Mechanisms of Action of Carbapenem Resistance. Antibiotics.

[12] Goravey W., Tsui C.K.M., Ali G.A., Najim M.S., Shunnar K., Ibrahim E.B., Ahmed M.A.S., Maslamani M.A., Sultan A., Skariah S. (2024). Clinical, phenotypic, and genotypic characteristics of ESBL-producing *Salmonella enterica* bloodstream infections from Qatar. IJID Reg..

[13] Fernandez J., Guerra B., Rodicio M.R. (2018). Resistance to Carbapenems in Non-Typhoidal *Salmonella enterica* Serovars from Humans, Animals and Food. Vet. Sci..

[14] Potter R.F., D’Souza A.W., Dantas G. (2016). The rapid spread of carbapenem-resistant Enterobacteriaceae. Drug Resist. Updat..

[15] Savard P., Gopinath R., Zhu W., Kitchel B., Rasheed J.K., Tekle T., Roberts A., Ross T., Razeq J., Landrum B.M. (2011). First NDM-positive *Salmonella* sp. strain identified in the United States. Antimicrob. Agents Chemother..

[16] Biswas S., Li Y., Elbediwi M., Yue M. (2019). Emergence and Dissemination of mcr-Carrying Clinically Relevant Salmonella Typhimurium Monophasic Clone ST34. Microorganisms.

[17] Wu Y., Jiang T., Bao D., Yue M., Jia H., Wu J., Ruan Z. (2023). Global population structure and genomic surveillance framework of carbapenem-resistant *Salmonella enterica*. Drug Resist. Updat..

[18] Grimont P.A.D., Weill F.-X. (2007). Antigenic Formulae of the Salmonella Serovars.

[19] Remel Europe Ltd (2023). Salmonella Agglutinating Sera. https://assets.fishersci.com/TFS-Assets/MBD/Instructions/X7823G.pdf.

[20] Clinical and Laboratory Standards Institute (CLSI) (2025). Performance Standards for Antimicrobial Susceptibility Testing.

[21] Sjolund-Karlsson M., Joyce K., Blickenstaff K., Ball T., Haro J., Medalla F.M., Fedorka-Cray P., Zhao S., Crump J.A., Whichard J.M. (2011). Antimicrobial susceptibility to azithromycin among *Salmonella enterica* isolates from the United States. Antimicrob. Agents Chemother..

[22] Hardy Diagnostics, NG Biotech Laboratories (2021). NG-Test CARBA 5. https://ngbiotech.com/amr-doc/Instructions_for_use_NG-Test-CARBA-5_for_the_United_States_v220125.pdf.

[23] Andrews S. FastQC: A Quality Control Tool for High Throughput Sequence Data. https://www.bioinformatics.babraham.ac.uk/projects/fastqc/.

[24] Beier S., Bolger A.M., Bolger M.E., Schwacke R., Usadel B. (2026). Trimmomatic: A decade of feature-rich, high-performance NGS read preprocessing. Bioinformatics.

[25] Prjibelski A., Antipov D., Meleshko D., Lapidus A., Korobeynikov A. (2020). Using SPAdes De Novo Assembler. Curr. Protoc. Bioinform..

[26] Gurevich A., Saveliev V., Vyahhi N., Tesler G. (2013). QUAST: Quality assessment tool for genome assemblies. Bioinformatics.

[27] Alikhan N.F. QualiBact: Species-Specific Quality Control of Bacterial De Novo Genome Assemblies. https://qualibact.org/.

[28] Jolley K.A., Bray J.E., Maiden M.C.J. (2018). Open-access bacterial population genomics: BIGSdb software, the PubMLST.org website and their applications. Wellcome Open Res..

[29] Public Databases for Molecular Typing and Microbial Genome Diversity. *Salmonella* spp.. https://pubmlst.org/organisms/salmonella-spp.

[30] Yoshida C.E., Kruczkiewicz P., Laing C.R., Lingohr E.J., Gannon V.P., Nash J.H., Taboada E.N. (2016). The Salmonella In Silico Typing Resource (SISTR): An Open Web-Accessible Tool for Rapidly Typing and Subtyping Draft Salmonella Genome Assemblies. PLoS ONE.

[31] Argimon S., Yeats C.A., Goater R.J., Abudahab K., Taylor B., Underwood A., Sanchez-Buso L., Wong V.K., Dyson Z.A., Nair S. (2021). A global resource for genomic predictions of antimicrobial resistance and surveillance of Salmonella Typhi at pathogenwatch. Nat. Commun..

[32] Jolley K.A., Maiden M.C. (2010). BIGSdb: Scalable analysis of bacterial genome variation at the population level. BMC Bioinform..

[33] Feng X., Li S., Huang D., Tan N., Li X., Xia S., Hu L., Cai R., Li Y., Wang J. (2025). Emergence of carbapenem-resistant XDR *Salmonella enterica* in paediatric patients in South China: A genomic perspective study. Int. J. Antimicrob. Agents.

[34] Zeng S., Huang Y., Zhang X., Fu L., Sun Z., Li X. (2023). Molecular characterization of IncFII plasmid carrying bla(NDM-5) in a *Salmonella enterica* serovar Typhimurium ST34 clinical isolate in China. mSphere.

[35] Li X., Jiang Y., Wu K., Zhou Y., Liu R., Cao Y., Wu A., Qiu Y. (2017). Whole-genome sequencing identification of a multidrug-resistant *Salmonella enterica* serovar Typhimurium strain carrying bla(NDM-5) from Guangdong, China. Infect. Genet. Evol..

[36] Yang L., Hu X., Xu X., Yang C., Xie J., Hao R., Du X., Wang L., Jia L., Li P. (2017). *Salmonella enterica* serovar Typhimurium ST34 co-expressing bla(NDM-5) and bla(CTX-M-55) isolated in China. Emerg. Microbes Infect..

[37] National Library of Medicine *Salmonella enterica* Strain 1722 Chromosome, Complete Genome. https://www.ncbi.nlm.nih.gov/nuccore/CP068018.1.

[38] Ke Y., Zhu Z., Lu W., Liu W., Ye L., Jia C., Yue M. (2024). Emerging bla (NDM)-positive *Salmonella enterica* in Chinese pediatric infections. Microbiol. Spectr..

[39] Ye J., Zheng Y., Hu D., Chen H., Jin Y., Ma Q., He J., Luo X. (2026). Tracing the evolutionary genomics of bla(NDM)-harboring *Salmonella typhimurium* in China. BMC Genom..

[40] Liu S., Zhang X., Xia J., Fang C., Zhao Y., Zhou M. (2026). Unveiling the Global Threat: Genomic Insights into Carbapenem-Resistant *Salmonella enterica* subsp. enterica in Human. Microb. Drug Resist..

[41] Gao Y., Wen J., Wang S., Xu X., Zhan Z., Chen Z., Bai J., Qu X., Zhang H., Zhang J. (2020). Plasmid-Encoded bla(NDM-5) Gene That Confers High-Level Carbapenem Resistance in Salmonella Typhimurium of Pork Origin. Infect. Drug Resist..

[42] Wang Z., He J., Li Q., Tang Y., Wang J., Pan Z., Chen X., Jiao X. (2020). First Detection of NDM-5-Positive *Salmonella enterica* Serovar Typhimurium Isolated from Retail Pork in China. Microb. Drug Resist..

[43] Wong T.K.F., Ly-Trong N., Ren H., Demotte P., Banos H., Roger A.J., Susko E., Bielow C., De Maio N., Goldman N. (2026). IQ-TREE 3: Phylogenomic inference software using complex evolutionary models. Mol. Biol. Evol..

[44] Bortolaia V., Kaas R.S., Ruppe E., Roberts M.C., Schwarz S., Cattoir V., Philippon A., Allesoe R.L., Rebelo A.R., Florensa A.F. (2020). ResFinder 4.0 for predictions of phenotypes from genotypes. J. Antimicrob. Chemother..

[45] Carattoli A., Zankari E., Garcia-Fernandez A., Voldby Larsen M., Lund O., Villa L., Moller Aarestrup F., Hasman H. (2014). In silico detection and typing of plasmids using PlasmidFinder and plasmid multilocus sequence typing. Antimicrob. Agents Chemother..

[46] Camacho C., Coulouris G., Avagyan V., Ma N., Papadopoulos J., Bealer K., Madden T.L. (2009). BLAST+: Architecture and applications. BMC Bioinform..

[47] Hong Kong Observatory Climate of Hong Kong. https://www.hko.gov.hk/en/cis/climahk.htm.

[48] Centre for Health Protection (2026). Review of Food Poisoning Outbreaks in Hong Kong, 2016–2025. Commun. Dis. Watch.

[49] Hornsey M., Phee L., Wareham D.W. (2011). A novel variant, NDM-5, of the New Delhi metallo-beta-lactamase in a multidrug-resistant *Escherichia coli* ST648 isolate recovered from a patient in the United Kingdom. Antimicrob. Agents Chemother..

[50] Peirano G., Chen L., Nobrega D., Finn T.J., Kreiswirth B.N., DeVinney R., Pitout J.D.D. (2022). Genomic Epidemiology of Global Carbapenemase-Producing *Escherichia coli*, 2015–2017. Emerg. Infect. Dis..

[51] Lian S., Liu C., Cai M., Cao Y., Xu X. (2024). Risk factors and molecular characterization of carbapenem resistant *Escherichia coli* recovered from a tertiary hospital in Fujian, China from 2021 to 2023. BMC Microbiol..

[52] European Centre for Disease Prevention and Control (2023). Increase in Escherichia coli Isolates Carrying blaNDM-5 in the European Union/European Economic Area, 2012–2022.

[53] Chu M.J., Zou M., Wang H., Wang J., You Z., Li Y., Liu B.T. (2026). Prevalence and genomic characterization of pathogenic bla(NDM)-positive *Escherichia coli* from retail meats: The first large-scale study in 22 cities of China. Food Microbiol..

[54] Cai Z.P., Zhu B.Q., Liu W.H., Hou J.Q., Wen X.E., He W.Y., Xiao L.D., Tu J.Y., Wu Z.W., Liu J.H. (2026). Outbreak and dissemination of the international high-risk NDM-5-producing *Escherichia coli* ST410 clone in companion animal hospitals in China. Zool. Res..

[55] Hu X., Xu X., Wang X., Xue W., Zhou H., Zhang L., Ma Q., Zhao R., Li G., Li P. (2017). Diversity of New Delhi metallo-beta-lactamase-producing bacteria in China. Int. J. Infect. Dis..

[56] Wu W., Feng Y., Tang G., Qiao F., McNally A., Zong Z. (2019). NDM Metallo-beta-Lactamases and Their Bacterial Producers in Health Care Settings. Clin. Microbiol. Rev..

[57] Xu B., Liu L., Fu H., Liu M., Li X. (2026). Cross-species transmission of bla (NDM-5)-IncX3 plasmids: Molecular mechanisms and One Health-based strategies for CREC control. Front. Cell Infect. Microbiol..

[58] Elnekave E., Hong S.L., Lim S., Boxrud D., Rovira A., Mather A.E., Perez A., Alvarez J. (2020). Transmission of Multidrug-Resistant *Salmonella enterica* Subspecies enterica 4,[5],12:i:- Sequence Type 34 between Europe and the United States. Emerg. Infect. Dis..

[59] De Sousa Violante M., Feurer C., Michel V., Romero K., Mallet L., Mistou M.Y., Cadel-Six S. (2024). Genomic diversity of Salmonella Typhimurium and its monophasic variant in pig and pork production in France. Microbiol. Spectr..

[60] Huang D., Wu Z., Jiang Y., Hu L., Cai R., Yang X., Du C., Chen S., Yang P., Guo B. (2025). Plasmid-driven clonal expansion of multidrug-resistant monophasic Salmonella Typhimurium in a Global Food Trade Hub. Emerg. Microbes Infect..

[61] Zhang K., Ge H., He J., Hu M., Xu Z., Jiao X., Chen X. (2022). Salmonella Typhimurium ST34 Isolate Was More Resistant than the ST19 Isolate in China, 2007–2019. Foodborne Pathog. Dis..

[62] Yu Z., Gunn L., Wall P., Fanning S. (2017). Antimicrobial resistance and its association with tolerance to heavy metals in agriculture production. Food Microbiol..

[63] Mourao J., Marcal S., Ramos P., Campos J., Machado J., Peixe L., Novais C., Antunes P. (2016). Tolerance to multiple metal stressors in emerging non-typhoidal MDR Salmonella serotypes: A relevant role for copper in anaerobic conditions. J. Antimicrob. Chemother..

[64] Li W., Li Y., Liu Y., Shi X., Jiang M., Lin Y., Qiu Y., Zhang Q., Chen Q., Zhou L. (2017). Clonal Expansion of Biofilm-Forming Salmonella Typhimurium ST34 with Multidrug-Resistance Phenotype in the Southern Coastal Region of China. Front. Microbiol..

[65] Research Office Legislative Council Secretariat (2016). Regulation of Imported Food in Hong Kong. https://www.legco.gov.hk/research-publications/english/1516fsc14-regulation-of-imported-food-in-hong-kong-20160226-e.pdf.

[66] Environment and Ecology Bureau Frequently Asked Questions on Food Supply of Hong Kong. https://www.eeb.gov.hk/food/download/press_and_publications/otherinfo/110318_food_supply_faq/e_food_supply_faq.pdf.

[67] Wang W., Baloch Z., Peng Z., Hu Y., Xu J., Fanning S., Li F. (2017). Genomic characterization of a large plasmid containing a bla (NDM-1) gene carried on *Salmonella enterica* serovar Indiana C629 isolate from China. BMC Infect. Dis..

[68] Peng J., Xiao R., Wu C., Zheng Z., Deng Y., Chen K., Xiang Y., Xu C., Zou L., Liao M. (2024). Characterization of the prevalence of Salmonella in different retail chicken supply modes using genome-wide and machine-learning analyses. Food Res. Int..

[69] Chen K., Peng J., Peng Z., Xiao R., Wang N., Deng Y., Wu C., Zheng Z., Guan J., Tang Y. (2025). Global genomic survey of Salmonella Kentucky: Discovery of a chromosomeborne bla(NDM-5) and the emergence of ST314, an MDR clone mediated by the IncR plasmid. Emerg. Microbes Infect..

[70] Wang J., Jiang Y., Mei C.Y., Wang Z.Y., Zhong F.G., Zhang X.X., Lv L.C., Lu M.J., Wu H., Jiao X. (2022). Characterization of an Extensively Drug-Resistant *Salmonella enterica* Serovar Indiana Strain Harboring Chromosomal bla (NDM-9) in China. Infect. Drug Resist..

[71] Hu Y.J., Huang P.Y., Tian M.S., Zheng L., He J., Li B.B., Zhao J.Y., Fanning S., Bai L., Dong Y.P. (2025). Genomic Epidemiology of Foodborne bla (NDM-9)Gene-Carrying Extensively Drug-Resistant (XDR) *Salmonella enterica* Serovar Indiana ST17. Biomed. Environ. Sci..

[72] Zhou Y., Wu K., Lin H., Hu Y.L., Xu W., Zhang J., Zhou S., Yu K., Liu C.G., Wang H.N. (2025). Whole genome sequencing of Salmonella in poultry from China reveals the presence of bla(NDM-5) in different serotypes. Poult. Sci..

[73] Wong I.T., Ng I.C., Cheung D.H., Tam W.Y., Wong C.Y., Fung S.K., Tong H.C., Chan S.K., Wu T.Y., Yeung E.Y. (2026). Genomic epidemiology and antimicrobial resistance of nontyphoidal Salmonella in retail meats in Hong Kong: A comprehensive surveillance study using whole-genome sequencing. Food Res. Int..

[74] Ho P.L., Wu T.C., Chau S.K.Y., Fong J.K.W., Lui L., Lung D.C., Tam A.R., Tang T.H.C. (2025). Reducing Bacterial Resistance with IMPACT.

[75] Ge B., Mukherjee S., Li C., Harrison L.B., Hsu C.H., Tran T.T., Whichard J.M., Dessai U., Singh R., Gilbert J.M. (2024). Genomic analysis of azithromycin-resistant Salmonella from food animals at slaughter and processing, and retail meats, 2011–2021, United States. Microbiol. Spectr..

[76] Chiou C.S., Hong Y.P., Wang Y.W., Chen B.H., Teng R.H., Song H.Y., Liao Y.S. (2023). Antimicrobial Resistance and Mechanisms of Azithromycin Resistance in Nontyphoidal Salmonella Isolates in Taiwan, 2017 to 2018. Microbiol. Spectr..

[77] Centre for Food Safety (2026). AMR Surveillance Findings. https://www.cfs.gov.hk/english/whatsnew/whatsnew_fstr/AMR_surveillance_findings.html.

[78] Centre for Health Protection (2026). Antimicrobial Resistance (AMR) Surveillance in Public Hospitals and Clinics—Hospital Authority AMR Data (2023). https://www.chp.gov.hk/en/static/109745.html.

[79] Centre for Health Protection Statistics on Antimicrobial Resistance Control. https://www.chp.gov.hk/en/statistics/data/10/100044/6971.html.

[80] World Health Organization (2024). WHO Updates List of Drug-Resistant Bacteria Most Threatening to Human Health. https://www.who.int/news/item/17-05-2024-who-updates-list-of-drug-resistant-bacteria-most-threatening-to-human-health.

[81] Simner P.J., Pitout J.D.D., Dingle T.C. (2024). Laboratory detection of carbapenemases among Gram-negative organisms. Clin. Microbiol. Rev..

[82] Rivera-Izquierdo M., Lainez-Ramos-Bossini A.J., Rivera-Izquierdo C., Lopez-Gomez J., Fernandez-Martinez N.F., Redruello-Guerrero P., Martin-delosReyes L.M., Martinez-Ruiz V., Moreno-Roldan E., Jimenez-Mejias E. (2021). OXA-48 Carbapenemase-Producing Enterobacterales in Spanish Hospitals: An Updated Comprehensive Review on a Rising Antimicrobial Resistance. Antibiotics.

