# Supplementary material for: Emergence of NDM-5-Positive Salmonella enterica Serovar Typhimurium Monophasic Variant ST34 in Hong Kong During 2020–2025
Source: Microorganisms. 2026 Oct 8;14(10):2289. doi: 10.3390/microorganisms14102289 (PMC13707440; doi:10.3390/microorganisms14102289)
Supplement: Supplementary file 1 [file microorganisms-14-02289-s001.zip › Supplementary Table S1_updated_20260917.pdf]

Supplementary Table S1. Antibiotics resistance genes detected in the 13 clinical carbapenem-resistant non-typhoidal *Salmonella* isolates included in this study

	SAL01	SAL02	SAL03	SAL04	SAL05	SAL06	SAL07	SAL08	SAL09	SAL10	SAL11	SAL12	SAL13
**Antimicrobial**	**Genetic background**	**Genetic background**	**Genetic background**	**Genetic background**	**Genetic background**	**Genetic background**	**Genetic background**	**Genetic background**	**Genetic background**	**Genetic background**	**Genetic background**	**Genetic background**	**Genetic background**
Gentamicin	*aac(3)-IV*	*ant(2'')-Ia*	*ant(2'')-Ia*	*aac(3)-IV*	*aac(3)-IV*, *rmtB*	Not found	Not found	*aac(3)-IId*	Not found	*aac(3)-IV*	*aac(3)-IV*	*aac(3)-IV*	Not found
Ciprofloxacin	*qnrS1*	*qnrS1*	*qnrS1*	*qnrS1*	*aac(6')-Ib-cr*	Not found	*qnrS1*	*qnrS1*	*qnrS1*	*qnrS1*	*qnrS1*	*qnrS1*	*qnrS1*
Amoxicillin	$bla_{\text{NDM-5}}$, $bla_{\text{OXA-10}}$, $bla_{\text{TEM-207}}$, $bla_{\text{TEM-30}}$, $bla_{\text{TEM-1B}}$	$bla_{\text{NDM-5}}$, $bla_{\text{CTX-M-55}}$	$bla_{\text{NDM-5}}$, $bla_{\text{CTX-M-55}}$	$bla_{\text{NDM-5}}$, $bla_{\text{OXA-10}}$, $bla_{\text{TEM-207}}$, $bla_{\text{TEM-30}}$, $bla_{\text{TEM-1B}}$	$bla_{\text{CTX-M-55}}$, $bla_{\text{NDM-9}}$, $bla_{\text{OXA-1}}$, $bla_{\text{TEM-206}}$, $bla_{\text{TEM-141}}$, $bla_{\text{TEM-1B}}$	$bla_{\text{NDM-5}}$, $bla_{\text{TEM-1B}}$	$bla_{\text{NDM-5}}$, $bla_{\text{TEM-1B}}$	$bla_{\text{NDM-5}}$, $bla_{\text{TEM-1B}}$	$bla_{\text{NDM-5}}$, $bla_{\text{TEM-1B}}$	$bla_{\text{NDM-5}}$, $bla_{\text{OXA-10}}$, $bla_{\text{TEM-207}}$, $bla_{\text{TEM-30}}$, $bla_{\text{TEM-1B}}$	$bla_{\text{NDM-5}}$, $bla_{\text{OXA-10}}$, $bla_{\text{TEM-207}}$, $bla_{\text{TEM-30}}$, $bla_{\text{TEM-1B}}$	$bla_{\text{NDM-5}}$, $bla_{\text{OXA-10}}$, $bla_{\text{TEM-207}}$, $bla_{\text{TEM-30}}$, $bla_{\text{TEM-1B}}$	$bla_{\text{NDM-5}}$, $bla_{\text{TEM-1B}}$
Sulfamethoxazole	*sul3*	*sul1*	*sul1*	*sul3*, *sul2*	*sul2*, *sul1*	*sul2*	*sul1*, *sul2*, *sul3*	*sul1*	*sul2*, *sul3*	*sul3*, *sul2*	*sul3*	*sul2*	*sul1*, *sul2*
Trimethoprim	*dfrA14*	Not found	Not found	*dfrA14*	*dfrA12*	Not found	*dfrA12*	*dfrA14*, *dfrA12*	*dfrA12*	*dfrA14*	*dfrA14*	*dfrA14*	*dfrA14*
Tetracycline	*tet(A)*	*tet(B)*	*tet(B)*	*tet(B)*, *tet(A)*	*tet(A)*	*tet(B)*	*tet(M)*, *tet(A)*	*tet(A)*	*tet(A)*, *tet(M)*	*tet(B)*, *tet(A)*	*tet(A)*	*tet(B)*, *tet(A)*	*tet(A)*
Azithromycin	Not found	Not found	Not found	Not found	*mph(A)*	Not found	Not found	Not found	Not found	Not found	Not found	Not found	Not found
Chloramphenicol	*floR*, *cmlA1*	Not found	Not found	*floR*, *cmlA1*	*floR*, *catB3*	Not found	*cmlA1*, *floR*	Not found	*cmlA1*, *floR*	*floR*, *cmlA1*	*floR*, *cmlA1*	*floR*, *cmlA1*	*floR*
Colistin	Not found	Not found	Not found	Not found	Not found	Not found	Not found	Not found	Not found	Not found	Not found	Not found	Not found
